# Development of A Questionnaire to Measure Attitude toward Oocyte Donation

**DOI:** 10.22074/ijfs.2015.4555

**Published:** 2015-10-31

**Authors:** Reza Omani Samani, Leila Mounesan, Zahra Ezabadi, Samira Vesali

**Affiliations:** Department of Epidemiology and Reproductive Health, Reproductive Epidemiology Research Center, Royan Institute for Reproductive Biomedicine, ACECR, Tehran, Iran

**Keywords:** Oocyte Donation, Attitude, Questionnaire, Infertility, ATOD-O

## Abstract

**Background:**

To our knowledge, there is no valid and comprehensive questionnaire that considers attitude toward oocyte donation (OD). Therefore this study has aimed to design and develop a tool entitled attitude toward donation-oocyte (ATOD-O) to measure attitude toward OD.

**Materials and Methods:**

This methodological, qualitative research was undertaken on 15 infertile cases. In addition, we performed a literature review and search of various databases. Validity of this questionnaire was conducted by knowledgeable experts who determined indices such as relevancy, clarity, and comprehensiveness. Reliability of the questionnaire was assessed based on the opinions of experts and infertile couples referred to Royan Institute.

**Results:**

ATOD-O was designed in 52 statements that covered various issues such as the OD process, donor and recipient characteristics, as well as family, emotional, psychological, legal, religious, and socio-economic dimensions. Results were scored as five points: 1 (strongly disagree), 2 (disagree), 3 (somewhat), 4 (agree), and 5 (strongly agree). The overall relevancy of the questionnaire was 97% and clarity was 96%. Overall comprehensiveness was 100%.

**Conclusion:**

The findings from this preliminary validation study have indicated that ATOD-O is a valid measure for measuring and assessing attitude toward donated oocytes. This questionnaire can be used in studies regarding different groups of a society.

## Introduction

There has been significant progress worldwide in the development of assisted reproductive techniques (ARTs) to aid infertile couples in achieving their reproductive goals (1). One of these techniques is oocyte donation (OD), initially introduced by Buster. The first infant was born via OD in 1984 (2).

OD is the process of ovulation stimulation in which a woman other than the infertile female partner donates her oocyte for fertilization. The donated oocyte is fertilized by the sperm of the infertile woman’s partner in the laboratory, after which the fertilized oocyte is subsequently transferred to the uterus of the infertile female partner (3). OD is a remarkably effective method of treatment, even in difficult cases (4). Challenges and problems associated with OD exist, such as disclosure of a child’s genetic origin and other ethical issues (5- 8). However this is the only way for infertile women who lack normal or high quality oocytes due to increased age, early menopause, birth defects, and genetic mutations, as well as chemotherapy and radiotherapy cancer treatments, despite the health of their other reproductive organs (9). The number of families that have been treated by this method is increasing. In the United States, the pregnancy rate through OD has reached 50% and the live birth rate has approximated this rate (10). A clear, exact and accurate rate for OD does not exist in Iran.

According to the Theory of Planned Behavior (TPB), attitude towards any behavior is one of the factors that help predict intentions to perform a given behavior (11). Therefore, it is necessary to measure and assess attitudes toward OD in the general population or other groups in a society. At present, OD is performed in Iran. To our knowledge, there is valid, comprehensive questionnaire regarding attitude toward OD. This study aims to design and develop a tool entitled attitude toward donation-oocyte (ATODO) to measure attitude toward OD.

## Materials and Methods

This methodological research was performed to design and develop a questionnaire with a Likert type scale to assess attitudes toward OD among infertile couples, donors and recipients of oocytes, and general population. This was a part of a big research entitled " attitude toward donation and surrogacy".

### Designing and developing attitude statements about oocyte donation

#### Qualitative research

We conducted a qualitative study in order to obtain attitude scale-items. Infertile couples referred to Royan Institute were included in this research using the quota method that took into consideration socioeconomics, age, and educational levels of the patients. Data saturation was accomplished after 12 couples. For assurance, we continued the interviews for a total of 15 couples. Content analysis was performed by two different researchers (M.Sc. and Ph.D. in Epidemiology) for better validity (member check).

#### Literature review

In order to identify the presence of an existing questionnaire, influencing factors, and other aspects on attitudes towards OD, we searched Iranian and international databases that included Magiran, Google Scholar, Science Direct, PubMed, and Iran Medex. Both internal and external related papers were studied. Therefore, other possible questions that related to any aspect of OD were designed. The questions were comprehensive to the best extent possible.

### Face validity

This type of validity indicates whether a test is apparently valid for subjects, administrative factors, and untrained observers (12). The face validity of ATOD-O has been assessed by 10 experts familiar and unfamiliar with the donation process. Experts took into consideration the proper sequence of questions, simple and illustrative form of the questionnaire, grammar, syntax, organization, appropriateness, and logical sequence of the statements (13).

### Content validity

Content validity determines the extent to which the questions of the tool are related to the objectives studied (14). In order to assess and evaluate content validity of this questionnaire, we have used 16 knowledgeable experts that included obstetricians and gynecologists (5 persons) and community medicine specialists (5 persons), as well as experienced managers, nurses, and experts familiar with the process of OD (6 persons). These experts determined indices such as relevancy (power and ability of statements that reflect content characteristics), clarity (clarity in correct spelling and statements’ concepts), and comprehensiveness (the ability of this tool to cover all relevant areas studied). The indices were subsequently assessed and assigned scores from 1 to 4, where a score of 1 was inappropriate, scores 2 and 3 were considered partly inappropriate and appropriate, and score 4 was quite appropriate. These individuals were asked to modify the statements they considered inappropriate. It should be said that the inter-rater agreement (IRA) by experts wa s calculated for the indices as follows.

We determined IRA on clarity and relevancy by dividing the statements that all experts agreed were appropriate by the total number of statements. The acceptable ratio was considered 70%. To specify clarity and relevancy of each statement, the numbers of experts who determined the indices for each statement were divided by the total number of experts in the study (15). As well, to delimit the overall clarity of the questionnaire, a dichotomous option (appropriate and inappropriate) was considered for each statement after merging inappropriate or partly inappropriate, and appropriate or quite appropriate options. The mean was used to calculate the overall relevancy of this tool, in which the total relevancy of each question was divided by the total number of questions. The overall clarity of the questionnaire was also obtained using the mean. In various studies, appropriate relevancy/clarity of a new tool was considered to be at least 80%. The overall comprehensiveness of the questionnaire was obtained by dividing the numbers of experts who recognized comprehensiveness of the questionnaire as appropriate by the total number of experts.

### Reliability

In this study, since the statements were qualitatively produced, we assessed reliability of the questionnaire based on the opinion of experts and infertile couples. Therefore, the statements had no capability for measuring repeatability of the total score in pre- and post-tests by intraclass correlation (ICC) and internal consistency reliability, using Cronbach’s alpha (16-18).

### Data analysis

Statistical analysis was performed using SPSS (SPSS Inc., Chicago, IL, USA) version 18. The significant level was considered 0.05.

### Ethical issues

This study was approved by the Ethical Committee of Royan Institute. The main objective of study was explained to participants. Informed consent from participants was obtained. The questionnaire contained no identifying information.

## Results

### Questionnaire design

**Fig.1:**
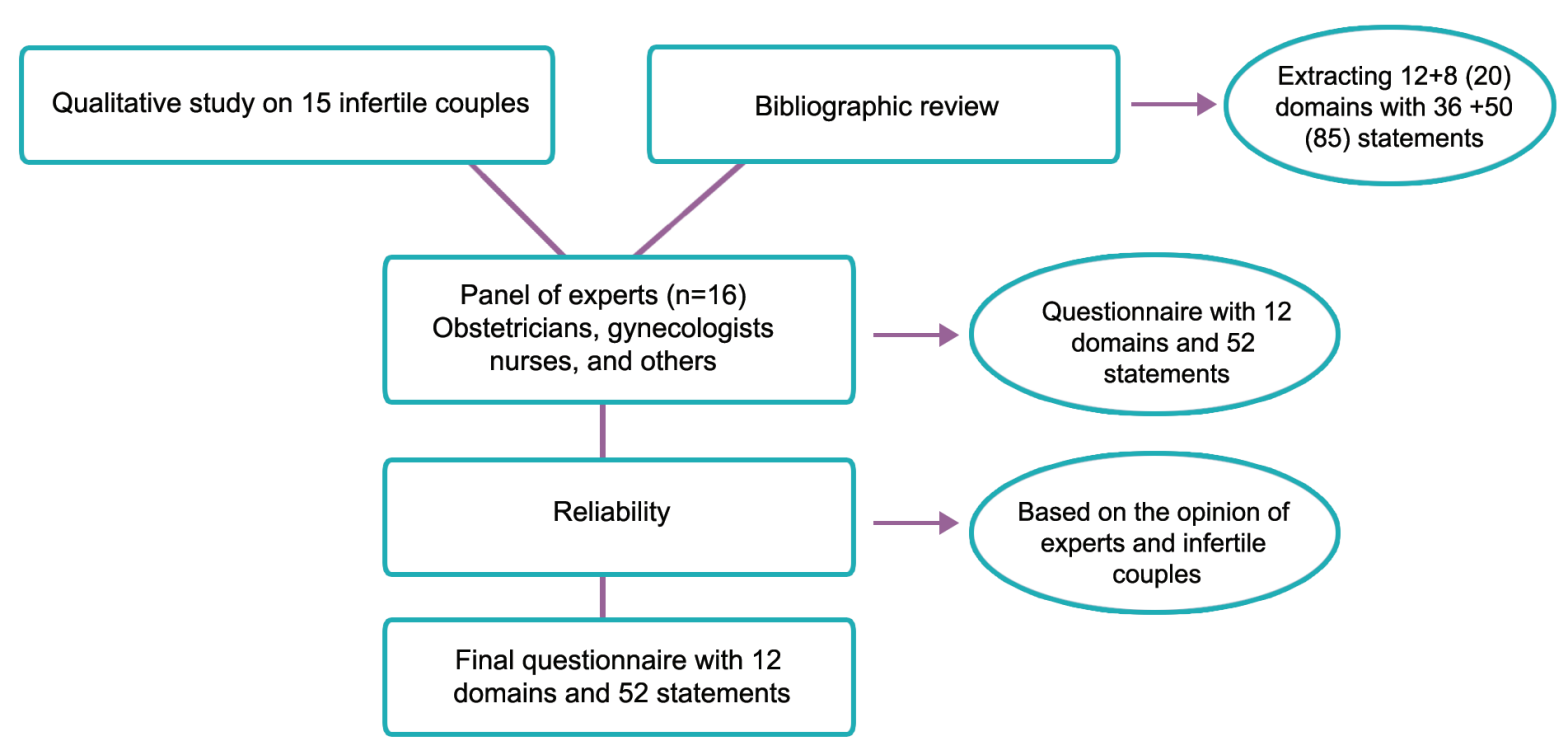
Flow chart related to the different stages of developing attitude toward donation-oocyte (ATOD-O).

We used data collected from the qualitative study and aspects obtained from database searches to generate a structured questionnaire. From the qualitative study, 12 domains were extracted from interviews and 8 domains were added from the literature review. After merging, deleting, and editing the items, they were reduced to 58 statements distributed in 12 domains. The different stages of the study and the outcomes obtained at each stage are shown in figure 1.

### ATOD-O questionnaire

In this study, we designed the questionnaire to include 58 statements according to various issues such as the OD process, donor and recipient characteristics, in addition to family, emotional, psychological, legal, religious, and socio-economic dimensions. According to the opinion of experts during content validity, we removed any unnecessary and less relevant questions. Some of the removed statements were as follows: "I agree to use oocytes from a living person"; the terms " IQ" and "morality" were deleted from "characteristics of the donor and recipient"; and "It is likely that the donor wants to see the child" was deleted. Finally, we reached a total number of 52 statements in 12 domains scored as follows: 1 (strongly disagree), 2 (disagree), 3 (somewhat), 4 (agree) and 5 (strongly agree). These domains included the importance of having children (2 statements), decision making and acceptance of OD (7 statements), playing the role of oocyte donor (5 statements), characteristics of the oocyte donor (8 statements), characteristics of the oocyte recipient (8 statements), being an anonymous child toward the donor (4 statements), disclosure of the use of this treatment method with others (3 statements), legal issues (4 statements), tendency to use different methods of OD (2 statements), the parent-child relationship (4 statements), and belonging of children (2 statements).

### Validity

Considering the opinion of experts in assessing content validity, 11 statements in 3 domains were also modified for clarity, relevance, and comprehensiveness. Additional details about modified statements are shown in table 1.

Findings indicated that the IRA on clarity was approximately 70% (36/52). The IRA on relevancy was 71% (37/52). The questionnaire had high overall relevancy (97%) and clarity (96%). The overall comprehensiveness of the questionnaire was 100%.

**Table 1:** Modified statements by experts during assessing content validity of attitude toward donation-oocyte (ATOD-O)


Domains	Statements

Decision making about receiving donated oocytes	I am ready to use oocyte donation if there is no any other therapy for infertility problem.
	Mental conditions of my male partner are important for receiving oocyte donated.
	Relatives or friends’ opinion is important for receiving oocyte donated for me.
	If my relatives or friends want to receive a donated oocyte, I would support their decision.
	Receiving oocyte donated is acceptable from my sister or relatives for me.
Decision making about donating oocytes	It is acceptable to give my oocyte my sister or relatives.
	I think that my male partner would agree on oocyte donation process for infertile couples.
	If my relatives or friends want to donate oocytes, I would support their decision.
Characteristics of an oocyte donor	The statement "beautiful appearance" was used instead of "a beautiful face".
	The statement "ethnicity and race" was used instead of "ethnicity".
	The statement "physical and mental health" was used instead of "physical health".


## Discussion

A systematic review on OD conducted in 2009 showed 64 eligible studies; most lacked standardized and validated questionnaires that did not report reliability and validity (2). The lack of valid and reliable questionnaires could lead to greater heterogeneity of the results in the review. Thus, a comparison of the studies made it difficult to reach a conclusion. Hence, this study was undertaken in order to develop and evaluate a new instrument for measuring attitudes toward OD. The instrument was primarily developed according to a qualitative study on 15 infertile couples to ensure that this new instrument would cover all existing concepts that pertain to OD. In addition, according to experts’ opinions, we removed any unnecessary and less relevant questions. The remaining questions were modified as statements. This tool included the following domains: OD process, donor and recipient characteristics, as well as family, emotional, psychological, legal, religious, and socioeconomic dimensions. We designed ATOD-O to be self-administered. However, in order to prevent selection bias due to illiterate participants and reduce missing data, this tool could also be used in an interview format.

Validity is requisite for a questionnaire because any defect or problem in the tool’s structure leads to bias and confounding results (19). Content validity is the first and most crucial step in a questionnaire design process, and a prerequisite for other validities. The validity improves the quality, and increases questionnaire reliability. In other words, reliability of a questionnaire is useless without content validity (20). In this study, we have determined the overall relevancy and clarity of ATOD-O to be higher than 0.9, which indicated appropriate validity. Obtaining feedback and opinions, and developing a tool by experts has been shown to enhance content validity (21). Therefore, the relatively high number of specialists involved in developing ATOD-O (16 specialists), despite the greater variance, was an advantage of this study due to high generalizability and agreement. The overall comprehensiveness of the questions was 100%. This suggested that important aspects related to the topic of interest were asked.

To measure reliability in quantitatively developed questionnaires, indexes such as ICC and Cronbach’s alpha are used. ICC assesses repeatability of the total questionnaire score by pre- and post-tests, whereas Cronbach’s alpha coefficient is applied to measure internal consistency (22-24). These indexes are used when questions from each domain in a tool have a correlation with each other (25, 26). In the current study, the statements have been obtained from the qualitative assessment, therefore they had a qualitative nature, but no correlation. No correlation was seen among statements of each item. Therefore, reliability of ATOD-O was assessed based on the opinion of experts and infertile couples.

We designed the statements to include both important aspects (psychological, scientific, and legal issues) and more general details. To increase external validity and generalization of the instrument, we applied the terms "female or male partners" instead of the words "wife or husband", respectively. In conservative or religious societies such as Islamic countries, laws and rights are consistent with the religious orders or recommendations obtained from religious establishments. As a result, cohabitation for couples is illegal and not permissible for non-married couples. Therefore, only married couples can undergo infertility treatments in these countries. If this tool is applied in such societies, it can be modified by taking into consideration legal issues.

Finally, ATOD-O can assess attitude toward OD in the general population, donors and recipients of oocytes, infertile couples, and other groups in a society. It is necessary to update questions over time because this technique (OD) may be used more frequently in the future and information about OD will increase among individuals and the general population.

## Conclusion

The findings from this preliminary validation study have indicated that ATOD-O is a valid tool for measuring and assessing attitude toward OD. It can be used in studies on different groups in a society. This newly developed scale can also be particularly useful and helpful to health professionals and authorities in order to assess the beliefs and attitudes of individuals regarding the OD process.
